# The Mechanisms of Bushen-Yizhi Formula as a Therapeutic Agent against Alzheimer’s Disease

**DOI:** 10.1038/s41598-018-21468-w

**Published:** 2018-02-15

**Authors:** Haobin Cai, Yunxia Luo, Xin Yan, Peng Ding, Yujie Huang, Shuhuan Fang, Rong Zhang, Yunbo Chen, Zhouke Guo, Jiansong Fang, Qi Wang, Jun Xu

**Affiliations:** 1 Institute of Clinical Pharmacology, Guangzhou University of Chinese Medicine, Guangzhou, 510405 China; 2 Research Center for Drug Discovery, School of Pharmaceutical Sciences, Sun Yat-sen University, Guangzhou, 510006 China; 3 Department of Neurology & Psychology, Fourth Clinical Medical College, Guangzhou University of Chinese Medicine, Shenzhen, 518033 China

## Abstract

Bushen-Yizhi prescription (BSYZ) has been an effective traditional Chinese medicine (TCM) prescription in treating Alzheimer’s disease (AD) for hundreds of years. However, the underlying mechanisms have not been fully elucidated yet. In this work, a systems pharmacology approach was developed to reveal the underlying molecular mechanisms of BSYZ in treating AD. First, we obtained 329 candidate compounds of BSYZ by in silico ADME/T filter analysis and 138 AD-related targets were predicted by our in-house WEGA algorithm via mapping predicted targets into AD-related proteins. In addition, we elucidated the mechanisms of BSYZ action on AD through multiple network analysis, including compound-target network analysis and target-function network analysis. Furthermore, several modules regulated by BSYZ were incorporated into AD-related pathways to uncover the therapeutic mechanisms of this prescription in AD treatment. Finally, further verification experiments also demonstrated the therapeutic effects of BSYZ on cognitive dysfunction in APP/PS1 mice, which was possibly via regulating amyloid-β metabolism and suppressing neuronal apoptosis. In conclusion, we provide an integrative systems pharmacology approach to illustrate the underlying therapeutic mechanisms of BSYZ formula action on AD.

## Introduction

Alzheimer’s disease (AD) is a progressive neurodegenerative disease that impairs memory and cognitive judgement. The pathological hallmarks of AD are characterized by the extracellular aggregation of amyloid-β peptides and intracellular inclusions of neurofibrillary tangles^1^. It has been reported that the number of people worldwide diagnosed with dementia will reach over 130 million by 2050 and cause tremendous social burdens. To date, acetylcholinesterase inhibitors (AChEIs), used as the first-line treatment for AD, provide only modest benefit clinically and might have potentially life-threatening side effects^2^. Moreover, approximately 200 clinical trials for AD therapies have been terminated due to ineffective treatment^3^. The strategy to reverse cognitive symptoms or progress with the presentation of a single treatment, targeting amyloid-β alone, may not be sufficient to treat this disorder. Thus, the development of effective therapeutic interventions for AD are urgently needed^4,5^.

Chinese herbal medicine, embracing centuries of knowledge and wisdom, has played a significant role in health maintenance in China^6^. Specifically, it offers effective ways to diagnose and treat chronic and degenerative diseases, include arthritis, diabetes and AD^7^. The purpose of TCM practice is to integrate a combination of herbs (Formula) based on the particular symptoms of patients and guided by the multiple theories of TCM^8^. Chinese medicine has been widely applied in the prevention and treatment of diseases for two thousand years and a number of TCM formulas are employed to improve cognitive function^9^. Bushen-Yizhi formula (BSYZ)^10^, an effective herb combination for AD treatment derived from the theories of TCM, consists of *Fructus Cnidii* (shechuangzi, SC), *Panax ginseng* (renshen, RS), *Polygonum multiflorum* (shouwu, SW), *Cortex moutan* (mudanpi, MD), *Ligustrum lucidum* (Nvzhenzi, NZ) and *Fructus lycii* (gouqizi, GQ). Our previous studies have revealed that BSYZ exerts anti-AD effects by modulating the cholinergic system and nerve growth factor signalling pathways, ameliorating oxidative stress and neuronal apoptosis *in vivo*^11,12^, protecting from scopolamine (SCOP)-induced cognitive impairment^13^, as well as ameliorating cognitive dysfunction through the SIRT1/endoplasmic reticulum (ER) stress pathway in ageing mice^14^. However, the bioactive substances and mechanisms of action (MOAs) of BSYZ on AD have not been identified and addressed, which impedes the modernization and clinical usage of BSYZ. Thus, it is necessary to identify the active ingredients of BSYZ, understand their synergistic actions in and the exact effects on multiple targets^15^.

**Figure 1:**
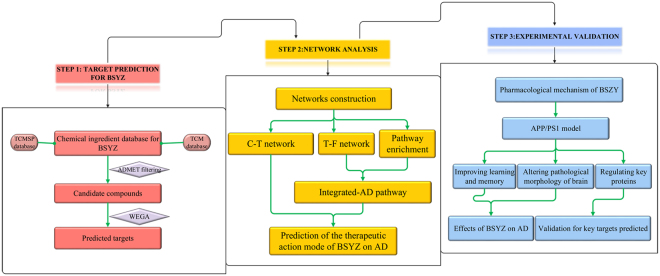
Flowchart of the systems pharmacology approach for uncovering the pharmacological mechanisms of Bushen-Yizhi Formula (BSYZ) actions on AD by integrating target identification, network analysis and experimental validation. (**A**) Target prediction for candidate compounds in BSYZ, (**B**) Network analysis to explore the therapeutic mechanisms of BSYZ on AD. (**C**) Experimental validation *in vivo* to decipher the pharmacological mechanisms of BSYZ on AD.

Systems pharmacology^16–18^, aiming to support drug discovery and clinical practice for the treatment of diverse complex diseases, has received much attention in recent years. As a novel strategy to identify the active compounds of herbs and their therapeutic targets, systems pharmacology has been used to facilitate a comprehensive understanding of the MOAs of Chinese herbal medicines^19–22^. In this study, we employed a systems pharmacology approach to investigate the active ingredients, potential targets and therapeutic MOAs of BSYZ in AD treatment (Fig. 1). We studied the compounds derived from BSYZ that met ADME/T requirements with Discovery Studio and an *in-house* pharmacophore shape comparison algorithm (WEGA)^23^. Then, the predicted targets were mapped into AD-relevant databases to determine their biological functions and corresponding AD pathways. Based on these results, we constructed networks to illuminate the MOAs of BSYZ on AD. Finally, we used an APP/PS1 mouse model to validate the proposed mechanisms for BSYZ as an anti-AD agent.

## Results and Discussion

### Putatively active moieties of BSYZ

A total of 688 chemical moieties derived from the BSYZ formula were collected from traditional Chinese medicine systems pharmacology (TCMSP)^24^ and traditional Chinese medicine (TCM)^25^ databases (duplicated compounds were removed). Among these compounds, 33 compounds were obtained from the Herbal Ingredients’ Targets (HIT) database^26^ and used as key herb ingredients (Supplementary Table S1).

By *in silico* ADME/T filtering, 329 candidate compounds out of 688 were predicted to be potential active compounds with appropriate pharmaceutical properties after filtering the compounds violating ADME/T rules. The number of the putative active compounds from SC, RS, SW, MD, NZ and GQ was 88, 98, 7, 15, 54 and 67, respectively. The structural information for the 329 compounds can be reviewed in Supplementary Table S2.

### Putative targets for the active compounds of BSYZ

With the WEGA algorithm, 766 targets were predicted for the 329 compounds. Among the predicted targets, 156 (20.37%) are associated with AD and involved in 276 compounds. Specifically, the number of potential AD-related targets connected by SC, RS, SW, MD, NZ and GQ was 149, 146, 120, 135, 146 and 142, respectively. Finally, we also identified 138 AD-related targets for 17 herb ingredients in BSYZ. The detailed information for putative AD-related targets is provided in Supplementary Table S3.

### Analysis of synergistic action of candidate targets in BSYZ

“Jun-Chen-Zuo-Shi” is also known as the “sovereign-minister-assistant-courier” principle^27^. In the theory of Chinese medicine, the Jun (emperor) herb is used to treat the main cause or primary symptoms of disease, while the Chen (minister) herb serves to augment or broaden the effects of Jun^28^. Except for MD, the other five herbs in the BSYZ Formula belong to Jun-Chen class of herbs. Herein, we investigated the distribution of targets for these five distinct herbs (149 from SC, 146 from RS, 120 from SW, 146 from NZ and 142 from GQ,) using InteractiVenn^29^.

**Figure 2:**
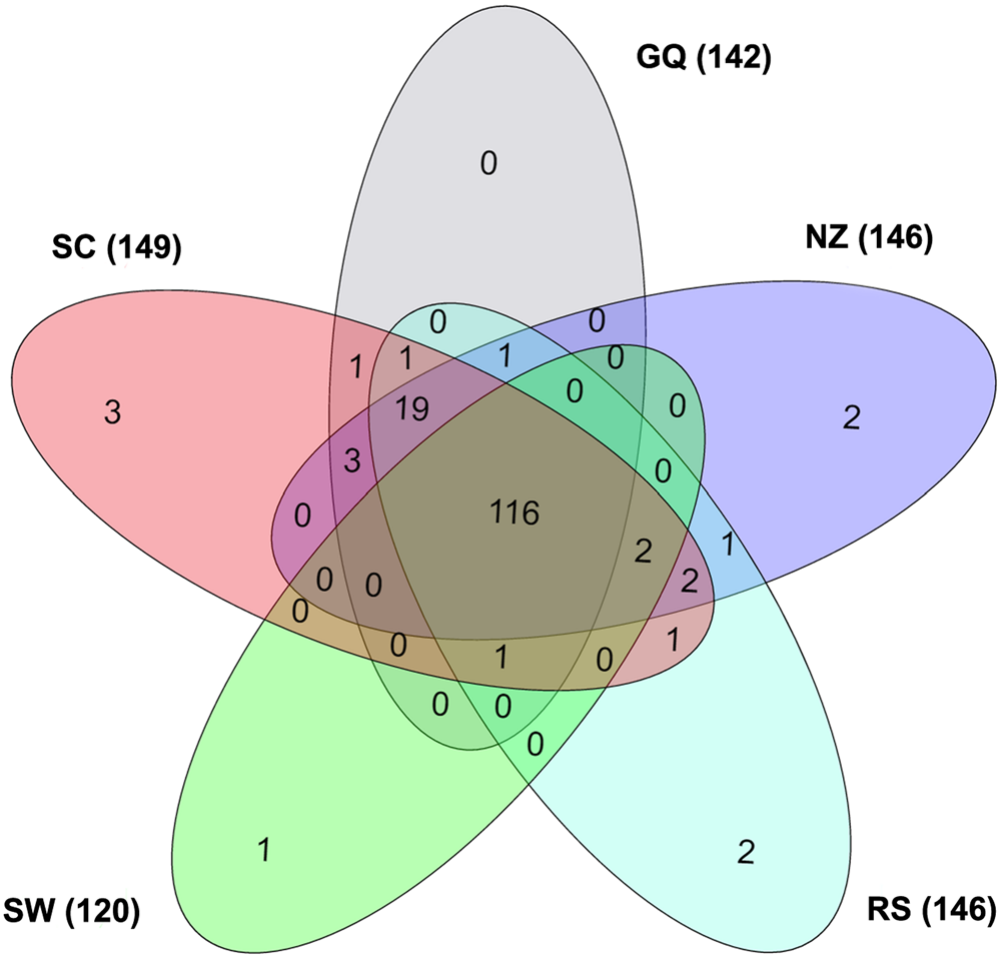
Overlapping candidate targets among five herbs in the Bushen-Yizhi Formula. The corresponding number of targets for each herb is 149 (Shechuangzi, SC), 146 (Nvzhenzi, NZ), 142 (Gouqi, GQ), 146 (Renshen, RS) and 120 (Shouwu, SW).

As Fig. 2 shows, the five herbs cover 156 targets in total. Interestingly, 116 out of them are common target proteins that exist in all of these five herbs simultaneously, indicating that BSYZ implements its magnifying effects based on common targets. These targets are calcium-dependent kinases (CAMK2A and CAMK2G), apoptosis/anti-apoptosis kinases, metalloelastases (MMPs), glutamate receptors and nerve growth factor receptors. Cathepsin D (CTSD), an intracellular protease with beta-secretase-like properties, promotes the aggregation of beta amyloid peptide^30^. Butyrylcholinesterase (BCHE) plays important roles in the process of AD through the non-specific hydrolysis of acetylcholine, increasing the levels of the amyloid precursor protein and interacting with interleukin-1. They are in the pathogenic pathway for AD^31^. Fatty acid amide hydrolase (FAAH) is a specific enzyme regulating the endogenous cannabinoid system implicating in neuroprotective and anti-inflammatory procedures^32^. These targets are related to amyloid β (Aβ) aggregation or neuron loss. The herbs may improve an AD patient’s status by regulating these proteins. The detailed information of common targets is provided in Supplementary Table S4.

### Investigating the therapeutic mechanism of BSYZ for AD through multi-level data integration

To elucidate the mechanisms of action of BSYZ in AD treatment, compound-target network analysis, target-function network analysis and integrated pathways analysis were performed in this work.

#### Compound-Target network (C-T network)

**Figure 3:**
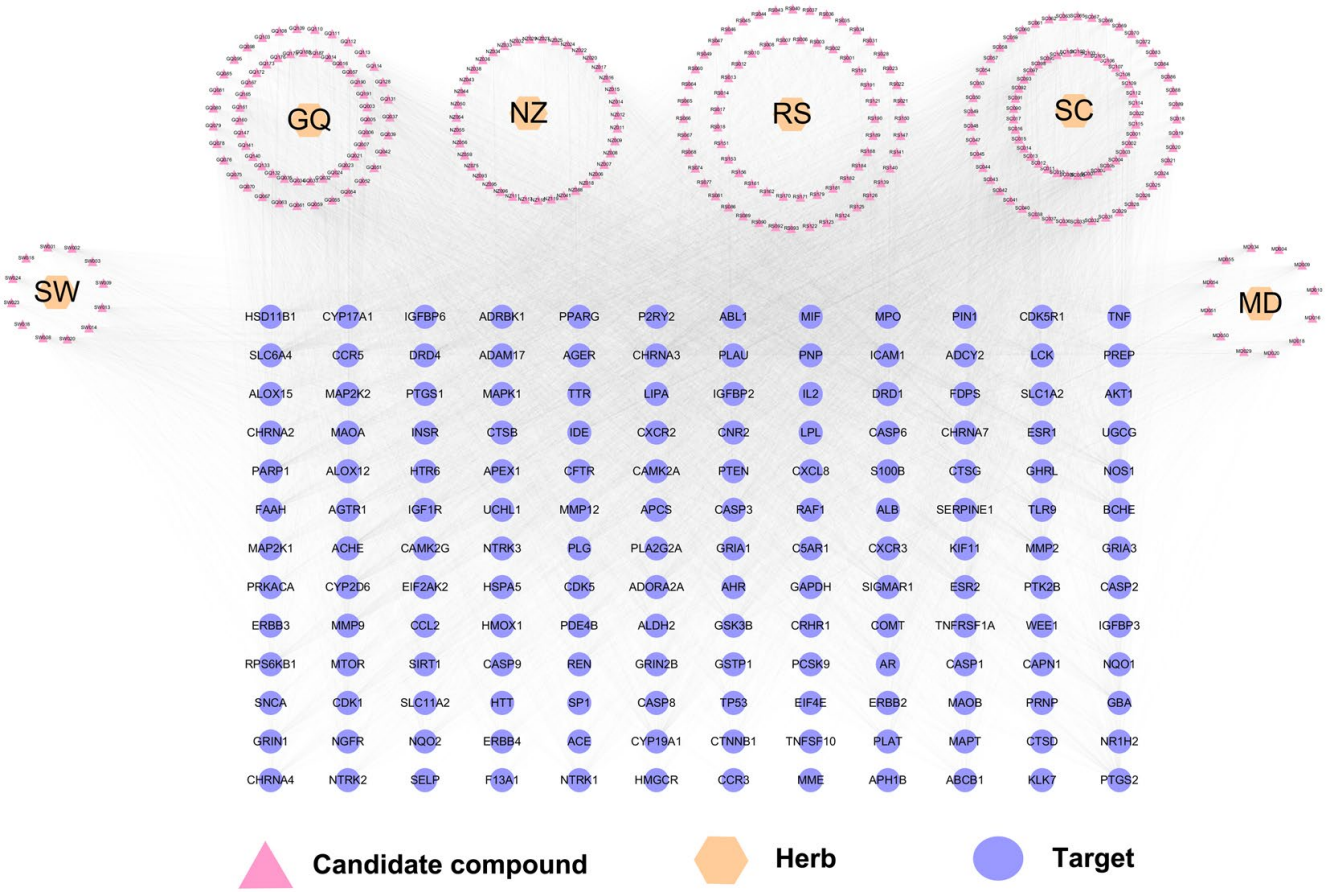
Global drug-target network of candidate compounds in the Bushen-Yizhi Formula. SC, Shechuangzi; NZ, Nvzhenzi; GQ, Gouqi; RS, Renshen; SW, Shouwu. MD, Mudanpi.

As shown in Fig. 3, the C-T network consists of 438 nodes (6 herbs, 276 compounds and 156 AD-related targets) and 13,914 C-T interactions, resulting in an average degree (the number of connections that each node has to other nodes) of 50.4 per compound and 89.2 per target.

**Figure 4:**
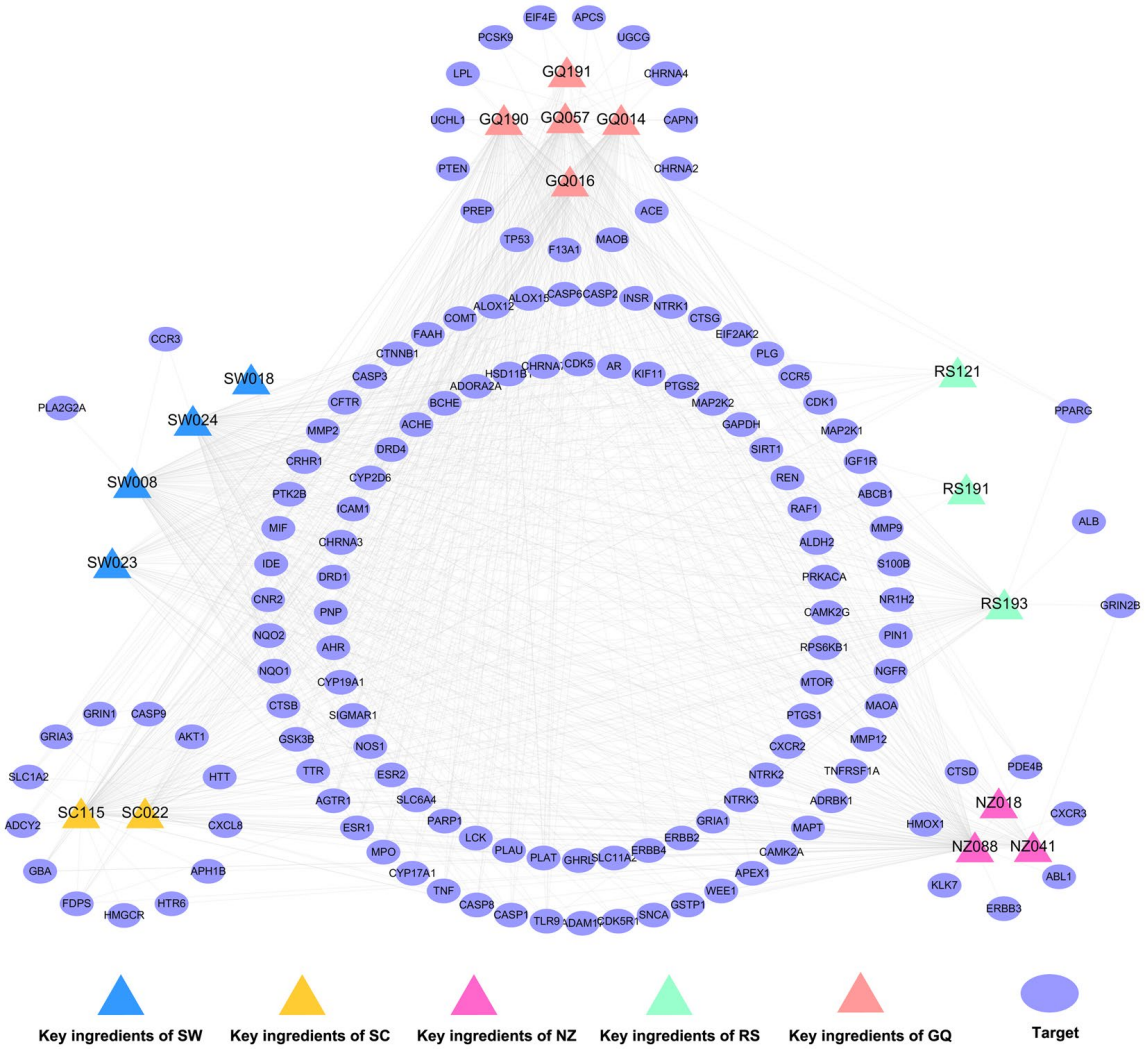
Specific drug-target network of key herb ingredients in the Bushen-Yizhi Formula. SC, Shechuangzi; NZ, Nvzhenzi; GQ, Gouqi; RS, Renshen; SW, Shouwu.

A C-T sub-network (Fig. 4) consists of 910 C-T pairs and 17 key ingredients connected with 138 AD-related targets. Among the 17 key ingredients, 13 of these possess a target degree (*K*) more than 15, particularly NZ088 (eriodicytol, *K* = 103), GQ057 (ascorbic acid, *K* = 100) and SW008 (physcion, *K* = 97), indicating that these ingredients play significant roles in the MOA of BSYZ. Among the targets, ACHE (acetylcholinesterase), SIGMAR1 (sigma non-opioid intracellular receptor 1) and SLC6A4 (sodium-dependent serotonin transporter) exhibited the highest connections (*N* = 13) to drugs, followed by CHRNA7 (neuronal acetylcholine receptor subunit alpha-7, *N* = 11) and NOS1 (nitric oxide synthase, brain, *N* = 11). All of these proteins are important in the pathogenesis of AD. ACHE is involved in regulating acetylcholine and can block cholinergic neurotransmission^33^. The activation of the sigma-1 receptor exhibits neuroprotective and neurorestorative actions for neurodegenerative diseases both *in vivo* and *in vitro*^34^. Thus, BSYZ may modify AD status by targeting these proteins.

#### Target-Function network (T-F network)

The T-F network demonstrates the relationships between AD-related biological processes and associated targets based on a DAVID analysis^35^.

**Figure 5:**
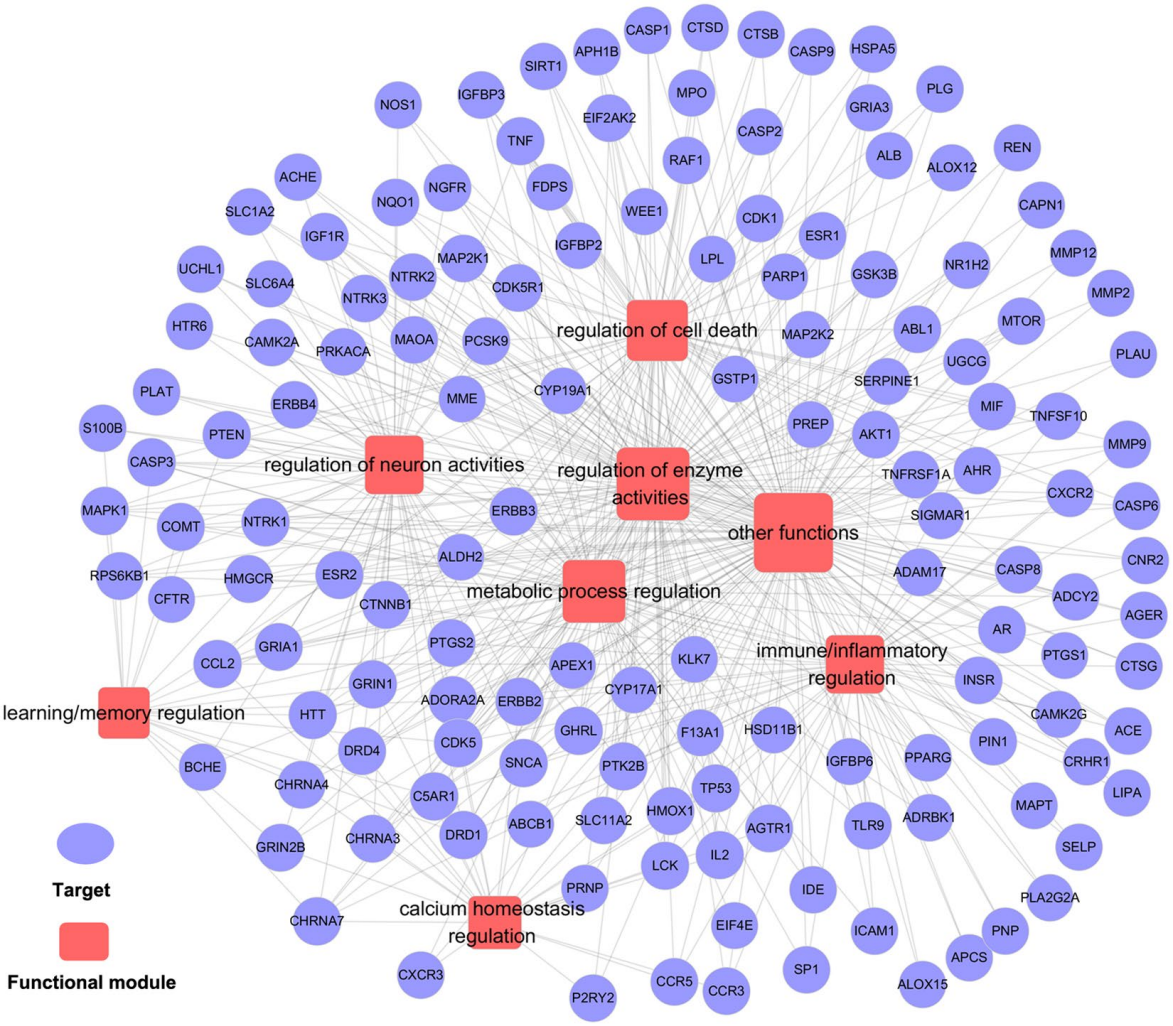
Target-function network. A functional module is linked to a target if the target is involved in that biological process.

As depicted in Fig. 5, this network consists of 516 target-function pairs connecting 145 targets with 8 AD-related functional modules. These modules consist of neuronal activities associated with learning and memory, enzymic activities, metabolic processes, immuno and inflammatory activities, calcium homeostasis and cell death. On average, a target is involved in 3.56 functional modules and 25 out of 145 targets are associated with more than 5 functional modules. Figure 5 indicates that BSYZ regulates calcium homeostasis via cellular/cytosolic calcium ion homeostasis and calcium ion transport. AD is associated with neuronal calcium-signalling abnormalities; therefore, restoring calcium signalling homeostasis is a strategy for anti-AD drug discovery^36,37^.

#### Pathway analysis

**Figure 6:**
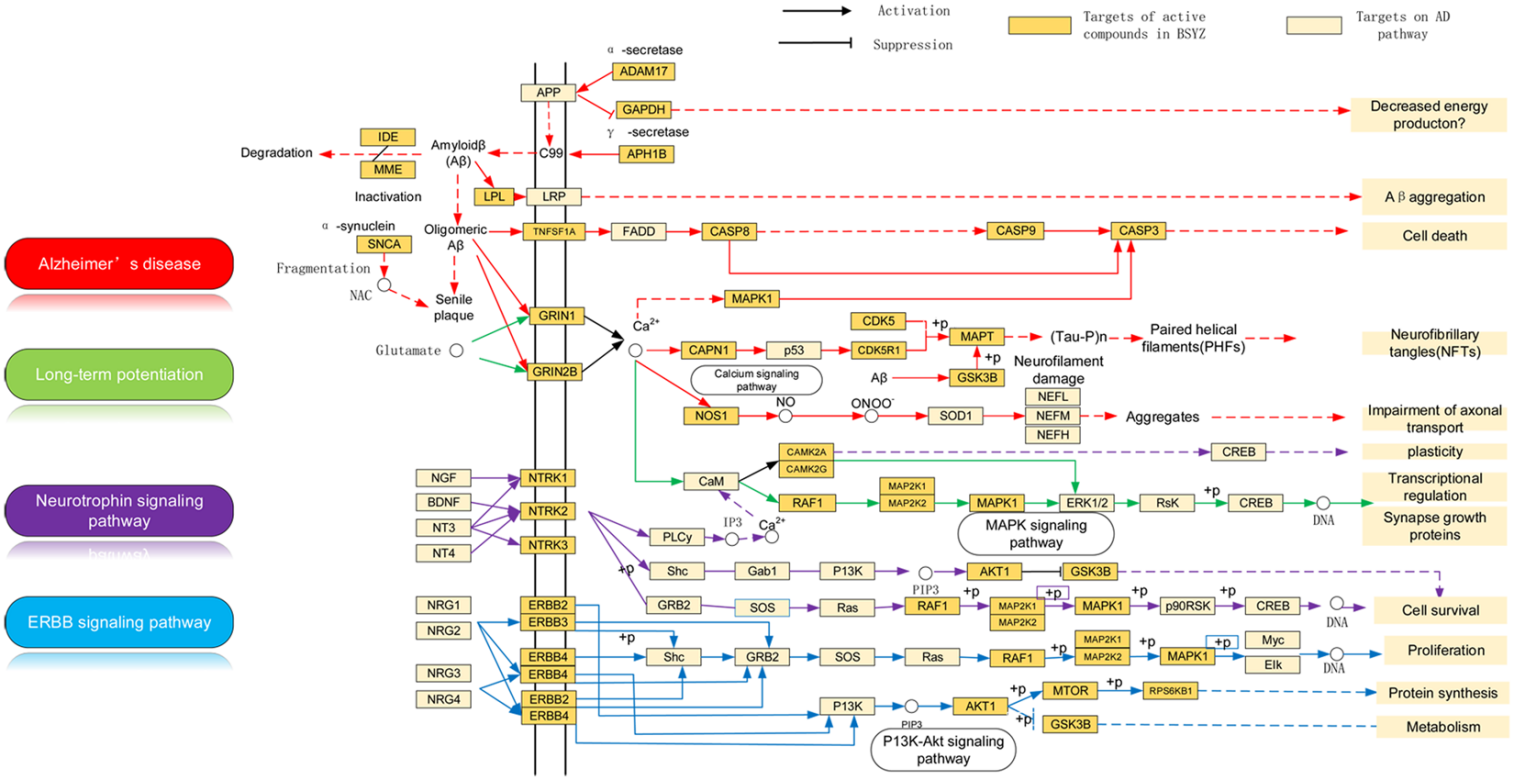
Integrated AD-pathway and therapeutic modules^64^. The orange nodes are potential protein targets for candidate compounds in the BSYZ Formula.

Pathways directly associated with AD were integrated into an “AD-pathway” network based on target prediction and AD pathology. The AD-pathway (Fig. 6) is divided into several pathological modules. Four representative modules reveal the underlying mechanisms of BSYZ as an anti-AD agent.

#### Aβ regulation module

Aβ aggregation is caused by neuronal dysfunction^38^ and is the successive proteolytic product of the precursor amyloid precursor protein (APP). Reducing neurotoxic Aβ generation is thought to be a promising therapeutic strategy. As shown in Fig. 6, the targets tagged with “AD pathway” are associated with the modulation of Aβ levels in AD patients. NZ044 and RS156 were predicted to stimulate ADAM17 (disintegrin and metalloproteinase domain-containing protein 17). ADAM17 was reported to modulate α-secretase effects on APP^39^. Targeting ADAM17 can change AD status by increasing the secretion of the neuroprotective soluble APPα fragment and reducing Aβ generation. In addition, both NZ088 and RS182 were predicted to bind with APH1B (gamma-secretase subunit APH-1B). The inhibition of APH1B can prevent APP cleavage to reduce Aβ production^40^. Furthermore, IDE (insulin-degrading enzyme) and MME (neprilysin, also known as NEP) play crucial roles in the pathological progress of AD by cleaving Aβ^41,42^. Compounds RS035 and NZ025 were predicted to improve IDE and MME activities, which could reduce Aβ aggregation in the brain. LPL(Lipoprotein lipase, also known as LIPL) has been shown as a novel Aβ-binding protein that promotes cellular uptake and subsequent degradation of Aβ^42,43^ and GQ016 and NZ012 predicted to regulate LPL for Aβ degradation could be a benefit for AD patients. Therefore, BSYZ might prevent and treat AD by regulating Aβ metabolism.

#### Neurofibrillary tangles (NFTs) regulation module

In an AD patient’s brain, NFTs are aggregated by abnormally hyperphosphorylated microtubule-associated protein tau (MAPT) and are considered to be one of the two main histopathological hallmarks of AD^43^. Therefore, targeting tau protein hyperphosphorylation provides a potential therapeutic avenue for AD treatment. As shown in Fig. 6, proteins targeted by BSYZ are implicated in the progression of NFTs. GQ191, NZ041, RS193, GQ190, SC115 and SW024 are predicted to interact with GSK3B (glycogen synthase kinase 3B) and CDK5 (cyclin-dependent kinase 5). Inhibiting these kinases can prevent NFI formation. p35 and CDK5/p35, which are CDK5 activators, can be cleaved by calpain (CAPN1)^44,45^. Candidate compounds were predicted to bind to CAPN1, suggesting the potential mechanism of BSYZ on preventing NFT formation.

#### Neurotrophin modulation module

Neurotrophic factors have attracted increasing attention for their potent neurogenesis effects and neuronal and synaptic plasticity^46,47^. Emerging evidence suggests that AD patients have imbalanced in brain-derived neurotrophic factor (BDNF) levels^48^. On the other hand, beta-nerve growth factor (NGF) exhibits activities of promoting the survival of cholinergic neurons in the brain^49^. BDNF, NGF and NT4 (neurotrophin 4), can selectively bind to different receptors to regulate neuronal survival, proliferation and differentiation^50,51^. NGF binds to high affinity nerve growth factor receptor 1 (NTRK1), while BDNF and NT4 bind to NTRK2. As shown in Fig. 6, the BSYZ formula regulates neurotrophin signal pathways to benefit individuals with AD by targeting various NTRKs. Compounds GQ057, NZ088 and RS193 interact with NTRK1, NTRK2 and NTRK3, which might improve the level of neurotrophins.

#### Long-term potentiation module

Long-term potentiation (LTP) is a putative mechanism of synaptic plasticity for learning and memory^52,53^. LTP is initiated by N-methyl D-aspartate (NMDA) receptors (e.g., GRIN1 and GRIN2B), followed by postsynaptic calcium influx and several protein kinase cascades (e.g., mitogen-activated protein kinases (MAPKs))^54^. In MAPK cascades, RAF1 stimulates the dual specificity mitogen-activated protein kinase kinase 1 (MAP2K1) and MAP2K2, which serves as an activator of MAPK1, resulting in LTP generation^55^. Therefore, LTP modulation and activation can improve learning and memory for AD patients. Figure 6 shows that BSYZ initiates and generates LTP via various LTP-related targets.

### BSYZ improved learning and memory abilities in APP/PS1 mice

Morris water maze (MWM) and novel-object recognition tests were used to evaluate the effects of BSYZ on learning and memory abilities of APP/PS1 mice.

**Figure 7:**
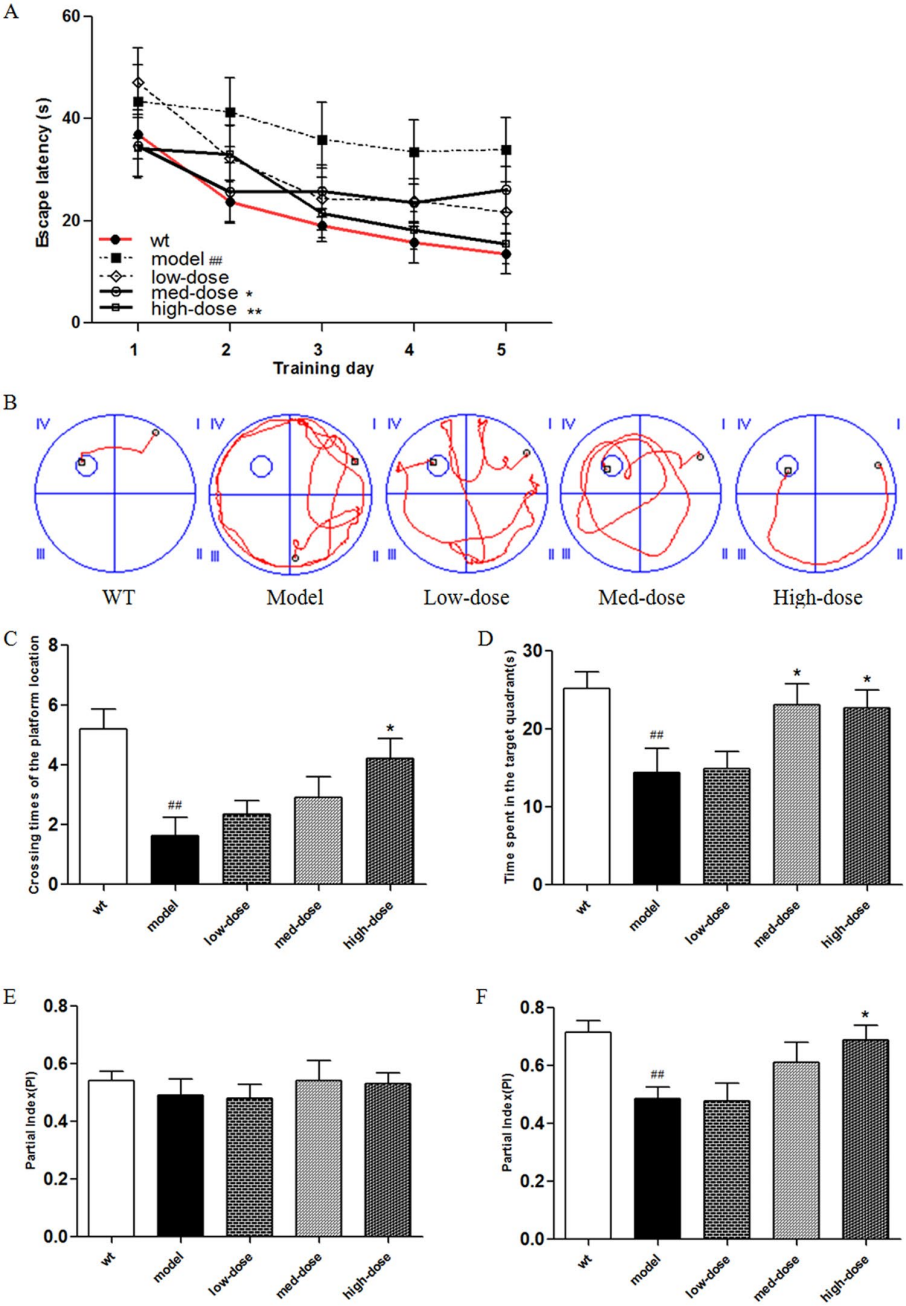
Bushen-Yizhi Formula improved learning and memory abilities in APP/PS1 mice. (**A**) Escape latencies in water maze during the positioning navigation test. (**B**) Representative tracings of animal paths during the positioning navigation test on the 5^th^ day. (**C**) Number of times crossing the platform location during the probe trial. (**D**) Time spent in the target quadrant and in the opposite quadrant during the probe trial. (**E**) The partial index on the second day during the novel-object recognition test. (**F**) The partial index on the third day during the novel-object recognition test. Groups included a low-dose of BSYZ (1.46 g/kg/d), a medium-dose of BSYZ (2.92 g/kg/d) and a high-dose of BSYZ (5.84 g/kg/d). Data are shown as the mean ± SD (n = 11 in each group). ^#^*P* < 0.05 and ^##^*P* < 0.01 versus the wild-type group. ^*^P < 0.05 and ^**^P < 0.01 versus the model group.

In the MWM test, the time for a mouse to find the hidden platform declined progressively over a 5-day positioning navigation test (Fig. 7A). As shown in Fig. 7A, compared with a wild-type group, the escape latency in the model group was significantly prolonged (*P* < 0.01 vs. wild-type), while the escape latency of the BSYZ medium-dose and high-dose groups was shorter compared with the model group (*P* < 0.05, *P* < 0.01, respectively. vs. model). All of these data were results of a 5-day repeated-measured analysis. Figure 7B presents representative tracings of animal paths during the positioning navigation test on the 5th day. As shown in Fig. 7B, compared with the model group, animals in the BSYZ groups spent less time finding the hidden platform. On the last day of MWM test, the platform was removed for a probe test. The number of times the animals crossed the platform position and the time spent swimming in the target quadrant are shown in Fig. 7C and D. The number of times the animals crossed the platform position in the model group was significantly less than that of the wild-type group (*P* < 0.01 vs. wild-type). The high-dose mouse group crossed significantly more times than the model group (*P* < 0.01 vs. model) (Fig. 7C). As shown in Fig. 7D, the mice in the model group spent significantly less time swimming in the target quadrant than the wild-type group (*P* < 0.01 vs. wild-type). The time spent swimming in the target quadrant for mice in the medium-dose and high-dose groups was significantly longer than that of the model group (*P* < 0.05, *P* < 0.05, respectively, vs. model).

In the novel-object recognition test, when mice were exposed to a box with the 2 same objects on the second day, there was no significant difference in the partial index between any two experimental groups (*P* > 0.05) (Fig. 7E). However, when one of the objects was replaced with a new one on the third day, the partial index in the model group was significantly lower in comparison to the wild-type group (*P* < 0.01 vs. wild-type) (Fig. 7F). The partial index in the high-dose group was significantly higher in comparison to the model group (*P* < 0.05 vs. model) (Fig. 7F).

These results prove that BSYZ improved the cognitive function of APP/PS1 mice.

### BSYZ decreased amyloid-β levels and ameliorated the senile plaques in APP/PS1 mice

**Figure 8:**
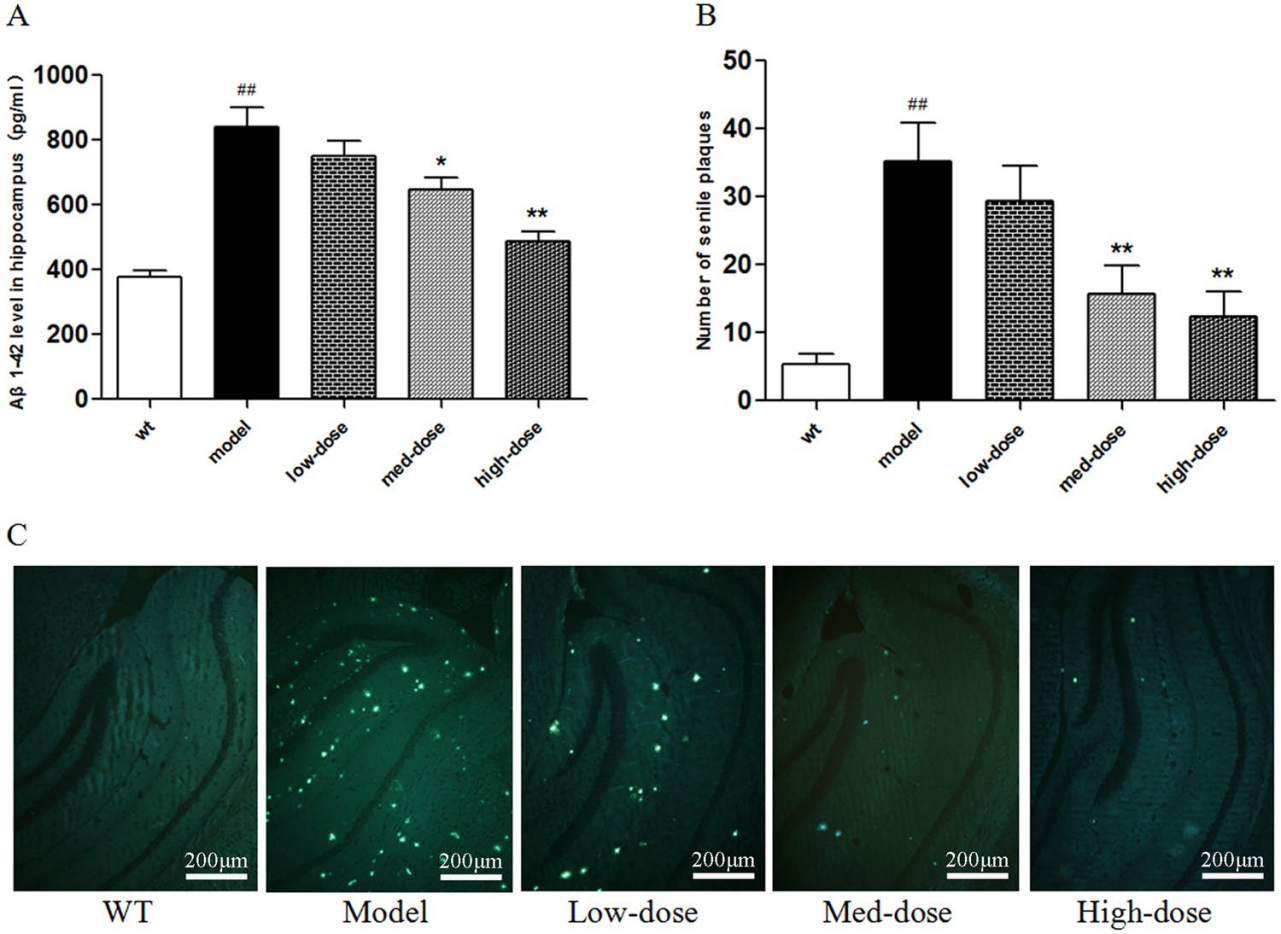
Bushen-Yizhi Formula decreased amyloid-β levels and ameliorated the senile plaques in APP/PS1 mice. (**A**) The level of Aβ_1–42_ in the hippocampus. (**B**,**C**) Quantitative graph (**B**) and representative images (**C**) of Thioflavine-S staining in the brains of APP/PS1 mice (magnification: 20x). Groups included a low-dose of BSYZ (1.46 g/kg/d), a medium-dose of BSYZ (2.92 g/kg/d) and a high-dose of BSYZ (5.84 g/kg/d). Data are shown as the mean ± SD (n = 11 in each group). ^#^*P* < 0.05 and ^##^*P* < 0.01 versus the wild-type group. *P < 0.05 and **P < 0.01 versus the model group.

We used ELISA to detect the amyloid-β_1–42_ (Aβ_1–42_) levels in the brains of all experimental mice (Fig. 8A). The model group presented significantly higher levels of Aβ_1–42_ compared with the wild-type group (*P* < 0.01 vs. wild-type) (Fig. 8A). More importantly, we found that the levels of Aβ_1–42_ in the BSYZ medium-dose and high-dose groups was significantly decreased compared with the model group (*P* < 0.05, *P* < 0.01, respectively, vs. model) (Fig. 8A). In addition, we used Thioflavine-S (Th-S) staining to further confirm the effect of BSYZ on the deposition of Aβ. As shown in Fig. 8B and C, there are extensive senile plaques present in the brains of APP/PS1 mice. The number of senile plaques was significantly decreased in the brains of mice from the BSYZ medium-dose and high-dose groups compared with the model group (*P* < 0.01, *P* < 0.01, respectively, vs. model) (Fig. 8B,C). These results indicate that BSYZ reduces amyloid-β generation and deposition in APP/PS1 mice.

### BSYZ regulates the amyloid-β metabolism and inhibits neuronal apoptosis in APP/PS1 mice

**Figure 9:**
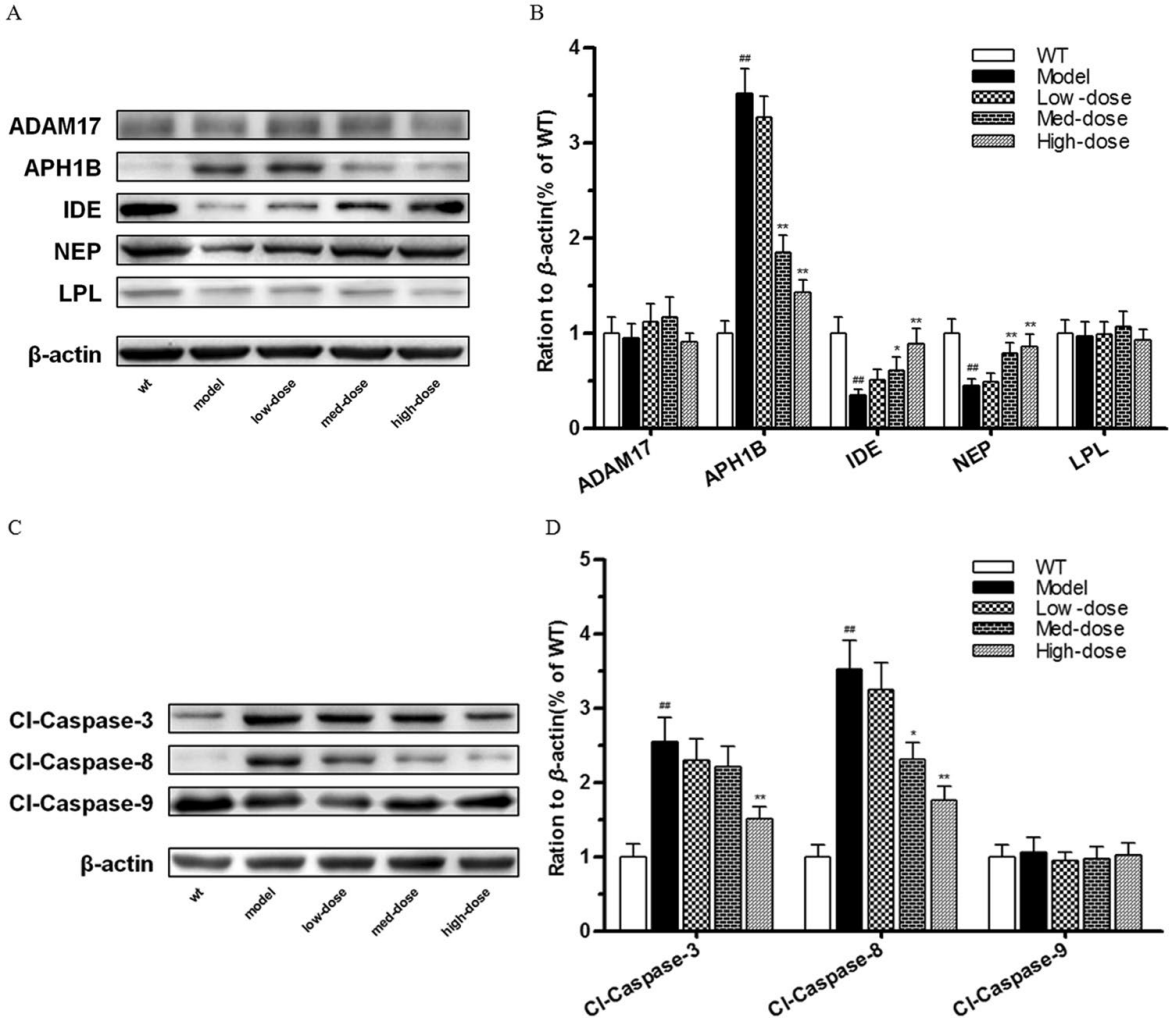
Bushen-Yizhi Formula regulated amyloid-β metabolism and inhibited neuronal apoptosis in APP/PS1 mice. (**A**,**B**) The levels of proteins associated with amyloid-β metabolism in the hippocampus. (**C**,**D**) The levels of proteins associated with neuronal cell apoptosis in the hippocampus. Groups included a low-dose of BSYZ (1.46 g/kg/d), a medium-dose of BSYZ (2.92 g/kg/d) and a high-dose of BSYZ (5.84 g/kg/d). Data are shown as the mean ± SD (n = 11 in each group). ^#^*P* < 0.05 and ^##^*P* < 0.01 versus the wild-type group. *P < 0.05 and **P < 0.01 versus the model group.

As discussed in Fig. 7, systems pharmacology-based investigations indicated that the therapeutic mechanism of BSYZ for AD involves various potential targets that regulate Aβ metabolism and neuronal apoptosis. To evaluate the effects of BSYZ on Aβ metabolism, we examined the expression levels of predicted target proteins including ADAM17, APH1B, IDE, NEP (MME) and LPL with Western blot experiments. With BSYZ treatment, no significant changes in ADAM17 and LPL among the five groups were found. However, BSYZ medium-dose and high-dose groups showed significantly decreased levels of APH1B (*P* < 0.01, *P* < 0.01, respectively, vs. model) in APP/PS1 mice (Fig. 9A,B). Moreover, the results showed that the expression levels of IDE and NEP in APP/PS1 mice were distinctly decreased compared with the wild-type group, yet increased by treatment with medium-dose BSYZ (*P* < 0.05 (IDE), *P* < 0.01 (NEP), vs. model) and high-dose BSYZ (*P* < 0.01 (IDE), *P* < 0.01 (NEP), vs. model) in a dose-dependent manner (Fig. 9A,B).

The protein levels of cleavage caspase-3, cleavage caspase-8 and cleavage caspase-9 were analysed to detect the effect of BSYZ on neuronal apoptosis-related proteins in APP/PS1 mice (Fig. 9C,D). We found that the BSYZ high-dose group showed significantly reduced levels of cleavage caspase-3 (*P* < 0.01 vs. model) and cleavage caspase-8 (*P* < 0.01 vs. model) compared with the model group (Fig. 9C,D).

These findings suggest that BSYZ treatment effectively modulates Aβ metabolism and inhibits neuronal cell loss in APP/PS1 mice. Overall, the results were consistent with systems pharmacology analyses.

## Conclusion

BSYZ, a TCM formula for AD treatment in China, can ameliorate cognitive dysfunction in AD mouse models. In this study, we developed an integrative systems pharmacology approach to uncover the pharmacological mechanisms of BSYZ against AD with a network pharmacology analysis and experimental validations. For the first time, we identified 329 BSYZ candidate compounds by *in silico* ADME/T filters and 276 of them were predicted to bind to 156 AD-related targets using WEGA. We further defined the molecular mechanisms of BSYZ against AD through multi-level data integration, including compound-target network analysis, target-function network analysis and integrated pathways analysis. These predictions were experimentally validated. The data prove that BSYZ improves the learning memory abilities in APP/PS1 mice by regulating Aβ metabolism and neuronal apoptosis.

## Material and Methods

### Compound database

A traditional Chinese medicine systems pharmacology platform (TCMSP) contains herbal ingredients, predicts targets and associated drug-target-disease networks and predicts pharmacokinetic properties for herbal ingredients^24^. We derived the chemical structures of five herbs (GQ, NZ, SC, MD and RS) from the TCMSP database and one herb (SW) from the TCM database^25^. To obtain the key ingredients of BSYZ, we extracted important chemical ingredients for each herb by retrieving six herb names from the Herbal Ingredients’ Targets (HIT) database. HIT is a comprehensive and fully curated database that collects important herbal ingredients with known protein target information^26^.

### ADME filtering

Pharmacokinetic properties play an essential role in drug discovery. In this work, the ADME (absorption, distribution, metabolism and excretion) descriptors module of Discovery Studio (DS) 4.0^56^ was used to filter out the ingredients with low pharmacokinetic properties. We removed the ingredients that satisfied one of the following criteria: (1) the solubility value was lower than −8 (extremely low); (2) the BBB_level value was equal to 3 (low penetrant); (3) the CYP2D6 property was ‘TRUE’; or (4) the absorption_level value was equal to 3 (very low absorption).

### Target identification for BSYZ

Identifying protein targets for candidate compounds in a Formula is critical for systems pharmacology-based drug discovery. We performed target identification via our *in house* three-dimensional similarity algorithm (WEGA). WEGA seeks protein targets for a given molecule based on experimental structural data by means of steric and static metric algorithms. Specifically, it superposes a compound structure on the binding pose of a known ligand from the dimensional coordinates of the query compound, which is docked into the postulated protein binding pocket if the superpositioning is successful. Detailed information on WEGA can be acquired in references^23,57^. In this study, the threshold value of similarity was set to 0.8.

### AD-related targets

AlzPlatform (http://www.cbligand.org/AD/) is an AD-specific chemogenomics knowledgebase, which consists of AD-related chemogenomics data including AD-related genes and proteins^58^. In this work, a total of 388 proteins associated with human AD were derived from AlzPlatform (Supplementary Table S5).

### GO enrichment and clustering analysis for AD-related targets

To elucidate the anti-AD therapeutic mechanisms of potential targets of BSYZ, we conducted a Gene Ontology (GO) enrichment analysis to classify the relevant biological process (BP) or functional features via mapping predicted AD-related targets to the DAVID database (https://david-d.ncifcrf.gov/)^35^, a web-accessible programme providing a comprehensive set of functional annotations to understand the biological meaning behind genes. In addition, we performed a functional annotation clustering analysis based on the GO and BP enrichment results and divided the BPs into several modules exhibiting different physiological functions. Only terms with *P* values less than 0.05 were applied. The detailed functional modules and associated proteins are listed in Supplementary Table S6.

### Network construction

To explore the multi-scale therapeutic mechanisms of the BSYZ formula in addressing AD, we constructed two types of networks, a compound-target network (C-T network) and target-function network (T-F network). In the networks, the nodes represent compounds/targets/functional modules and edges represent links between them.

To visualize C-T and T-F networks, Cytoscape3.2.1^59^ software was used in the present work. Cytoscape is an open source software used to depict biomolecular interaction networks.

### Pathway construction and analysis

An integrated “AD-pathway” was generated based on the current knowledge of AD pathology. The predicted BSYZ targets were mapped to KEGG and classified into different types of pathways. The pathways closely connected to AD were consolidated into an “AD-pathway” in light of the pathological and nearness analysis data.

### Experimental validation

The mice used in this work were approved by Guangzhou University of Chinese Medicine Animal Ethics Committee. The experiments were conducted in accordance with policies and procedures described in the Guidelines for the Care and Use of Laboratory Animals published by the National Research Council.

### Animals

Seven-month-old APPswe/PSEN1ΔE9 transgenic and age-matched wild-type mice were obtained from the Model Animal Research Center of Nanjing University. Male mice were used, weighing 30–35 g and housed at 20–25 °C with 60% relative humidity under controlled conditions (12 h light/dark cycle) with free access to standard rodent diet and water.

### Drugs and treatments

The herbs in the BSYZ formula were obtained from Guangxi Yifang Chinese Herbal Medicine Department. A voucher specimen (NO.20121209) was deposited at Guangzhou University of Chinese Medicine. The six raw herbs (SC, RS, SW, MD, NZ and GQ) were mixed in a ratio of 3:3:2:2:2:2. After extraction with methanol, the extract solution was dried to a powder. The quality analysis of the BSYZ formula was performed in our previous study^11^.

APP/PS1 mice were randomly divided into 4 groups, a model group (n = 11), a low-dose of BSYZ group (1.46 g/kg/d, n = 11), a medium-dose of BSYZ group (2.92 g/kg/d, n = 11) and a high-dose of BSYZ group (5.84 g/kg/d, n = 11). The same aged non-transgenic mice were selected for the wild-type group (n = 11). The wild-type and model groups were given purified water (10 ml/kg/d) via intragastric administration once daily for 4 weeks.

### Reagents

Antibodies included rabbit anti-ADAM17 (1:1000; Abcam: ab173579), rabbit anti-Aph1b (1:1000; Abcam: ab128335), rabbit anti-LPL (1:1000; Abcam: ab137821), rabbit anti-NEP (1:1000; Abcam: ab126593), rabbit anti-IDE (1:1000; Abcam: ab133561), rabbit anti-caspase-3 (1:500; Cell Signaling: #9662), rabbit anti-caspase-8 (1:1000; Abcam: ab138485) and mouse anti-β-actin (1:60000; Sigma: A5441). Enzyme-linked immunosorbent assay (ELISA) kits included the mouse amyloid beta peptide 1–42 (15.6 pg/mL/1000 pg/mL) Emax immunoassay kit (CUSABIO: Lot: R09013057).

### Morris water maze test

The Morris water maze test was performed from day 29 to day 35. A pool (diameter: 100 cm; height: 50 cm; depth of water: 30 cm) was filled with water (22 ± 1 °C). A circle platform (diameter: 8 cm; height: 29 cm) approximately 1 cm beneath the water surface was placed at the centre of a target quadrant in the pool, invisible from the surface of the water. The water in the pool was dyed white. Mice were released into the water and trained to find the platform within 60s. In the cases of failure, mice were guided to the platform artificially. Once reaching the platform, mice were allowed standing on the platform for 20s and were then moved into a cage beside an electric radiator until their fur was dried. Four timed trials were carried out every day for 5 days. The mice were gently released into the pool facing towards the wall randomly in each trial. The swimming trajectory of each mouse was recorded by a video camera and a tracking system. The probe trial was performed 24 hours after the 5-day positioning navigation test with the platform removed. Mice were allowed to swim for 60s in the pool. The mice swimming trajectories within 60s were systematically recorded and analysed^60^.

### Novel-object recognition test

The Novel-object recognition test was conducted with a previously described protocol^61^. Mice were gently placed in the experimental box for 5 minutes on day 1. On day 2, mice were gently placed in the experimental box with 2 of the same objects. On day 3, mice were gently placed in the experimental box where one of the objects had been replaced with a new one. Object exploration time was recorded when the mouse touched the object directly with its mouth, forepaws or nose. The preference index was calculated as the time spent on the new object divided by the cumulative time spent on both objects.

### Western blot analysis

Western blot analysis was carried out as previously described^10^. The BCA Protein Assay was used to determine the protein concentration. Forty micrograms of protein were dissolved in 4 to 15% Bio-Rad polyacrylamide gels and then transferred onto polyvinylidene fluoride (PVDF) membranes (Millipore, Germany). The membranes were blocked in Odyssey (LI-COR) blocking buffer for 1.5 hours at room temperature and then incubated overnight in the corresponding primary antibody. Then, the membranes were incubated with the corresponding secondary antibodies. Enhanced chemiluminescent immunoblotting (ECL, Bio-Rad, Japan) was used to visualize the membranes. Western blot quantification was performed using Image Lab software.

### Thioflavine-S staining

Thioflavine-S(Th-S), a fluorescent dye, is commonly used to stain senile plaques^12,62^. The brain tissues of mice were first fixed with paraformaldehyde then dehydrated and embedded in paraffin. The tissues embedded in paraffin were sectioned with a microtome and fixed on a slide. Sections were dehydrated and finally rehydrated in distilled water and stained with Mayer’s haematoxylin for 5 min. Then, slides were rinsed with running water for 1 min and immersed in a Th-S solution (1% Th-S in distilled water) for 5 min. Slices were immersed in 70% alcohol for 5 min then washed with distilled water 2 times and, finally, glycerol gelatin was used for cover-slipping. All slices were observed blindly by another investigator using an optical microscope (Olympus BX 41 microscope, 40x magnification) using Image-Pro Plus software to calculate the number of senile plaques.

### Enzyme-linked immuno sorbent assay

Mouse amyloid beta peptide (Aβ)_1–42_ in hippocampal and cortical homogenates were quantified using enzyme-linked immuno sorbent assay kits following the manufacturer’s instructions as described^63^. Samples were compared with Aβ_1–42_ standard curves to calculate the level of Aβ_1–42_ in extracts.

### Statistical analysis

Statistical analysis software SPSS 19.0 was used to analyse the data. The data are represented as the Mean ± SD. Repeated-measures analysis of variance was used to analyse the escape latency of the 5-day positioning navigation test. One-way analysis of variance (ANOVA) was used for multiple comparisons followed with the Dunnett’s post hoc test. The S-N-K test was used when there was homogeneity of variance and the rank sum test was used when the variance was not uniform. *P* < 0.05 was considered statistically significant.

## Electronic supplementary material

Supplementary Table S1

Supplementary Table S2

Supplementary Table S3

Supplementary Table S4

Supplementary Table S5

Supplementary Table S6
